# Quality of life and mood disorders of mild to moderate stroke survivors in the early post-hospital discharge phase: a cross-sectional survey study

**DOI:** 10.1186/s40359-023-01068-8

**Published:** 2023-01-31

**Authors:** Amy Waller, Kristy Fakes, Mariko Carey, Joshua Dizon, Kim Parrey, Michelle Coad, Rob Sanson-Fisher

**Affiliations:** 1 Health Behaviour Research Collaborative, College of Health, Medicine and Wellbeing, University of Newcastle, Callaghan, NSW 2308 Australia; 2 Hunter Medical Research Institute, New Lambton Heights, NSW 2308 Australia; 3 College of Health, Medicine and Wellbeing, Centre for Women’s Health Research, University of Newcastle, Callaghan, NSW 2308 Australia; 4 Clinical Research Design and Statistical Services, Hunter Medical Research Institute, New Lambton Heights, NSW 2305 Australia; 5 Port Macquarie Base Hospital, Mid North Coast Local Health District, Port Macquarie, NSW 2444 Australia

**Keywords:** Stroke, Quality of life, Depression, Anxiety

## Abstract

**Background:**

Exploring sociodemographic and disease characteristics that contribute to patient-reported outcomes can inform targeting of strategies to support recovery and adaptation to stroke. This study aimed to examine among a sample of stroke survivors at three months post-hospital discharge: (1) self-reported physical and mental health quality of life scores; (2) self-reported depressive and anxiety symptoms; and (3) sociodemographic and clinical predictors of these outcomes.

**Methods:**

This cross-sectional survey study recruited stroke survivors from eight hospitals in one Australian state. Adult survivors recently discharged from hospital stroke wards (within 3 months) were mailed a study information package and invited to complete a pen-and paper survey. Survey items assessed: quality of life (SF12v2), depression (PHQ-9), anxiety (GAD-7) and sociodemographic and clinical characteristics. Predictors were examined using multiple linear regression analysis.

**Results:**

Of the 1161 eligible patients who were posted a recruitment pack, 401 (35%) returned a completed survey. Participants reported a mean SF-12v2 Physical Composite Score (PCS) quality of life score of 44.09 (SD = 9.57); and a mean SF-12v2 Mental Composite Score (MCS) quality of life score of 46.84 (SD = 10.0). Approximately one third of participants (34%; n = 132) were classified as depressed (PHQ-9 ≥ 10); and 27% (n = 104) were classified as anxious (GAD-7 ≥ 8). Lower PCS was associated with being female, not employed and having a comorbid diagnosis of diabetes and atrial fibrillation. Lower MCS was associated with a history of transient ischemic attack (TIA). Males and those with higher levels of education, had greater odds of having lower depression severity; those with a history of TIA or diabetes had lower odds of having lower depression severity. Males had greater odds of having lower anxiety severity; those with a history of TIA had lower odds of having lower anxiety severity.

**Conclusion:**

Sub-groups of stroke survivors may be at-risk of poorer quality of life and psychological morbidity in the early post-discharge phase. These findings support the role of early identification and prioritisation of at-risk survivors at discharge, as they may require modifications to standard hospital discharge processes tailored to their level of risk.

## Introduction

Globally, the lifetime risk of having a stroke is 25% [1]. An estimated 387,000 Australians have experienced a stroke at some time in their lives [2], many of whom require assistance with day-to-day living activities [3]. However, studies suggest that ongoing rehabilitation needs of stroke survivors are inconsistently assessed [4], and post-discharge information and support required by survivors to adjust to life after stroke in the community is inconsistently provided [5]. Stroke survivors have also expressed feelings of desertion once they are discharged to the community.

There has been a move toward the administration of Patient-Reported Outcome Measures (PROMS) to obtain stroke survivors’ own perspective of their health, in addition to traditional clinical outcomes [6, 7]. This coincides with growing interest in outcomes such as mood and health-related quality of life following discharge from acute hospital care and rehabilitation services [8–10] given the increasing number of stroke survivors living in the community. Studies examining health related quality of life (HRQoL) and contributing factors over time, including in the early discharge phase [11], report that on average, the HRQoL of stroke survivors remains below that of the general population [12]. Furthermore, improvements in HRQoL achieved during rehabilitation may not be sustained [13]. Approximately one third of stroke survivors experience post-stroke depression [14], with other mood disorders such as anxiety also prevalent (10% [15] to 27% [16]). The need for further work to better understand the physical and mental health impacts of stroke [17] and achieve improvements in quality of life, particularly for those at risk of or experiencing post-stroke depression [14], has been highlighted. A review of PROMS for stroke survivor populations also indicated a need for further research using validated measures [6].

There is also emerging evidence of a relationship between early phase and longer-term outcomes for stroke survivors. One Australian study reported that stroke survivors’ quality of life in the early period following stroke was linked to the development of longer-term unmet needs at a median of 2 years post-stroke [8]. Symptoms of post-stroke depression reportedly occur more frequently within the first three months after stroke, and less frequently in later post-discharge phase [18]. Four trajectories of depression symptoms have been identified, including those with severe symptoms (6.31%) that may decrease slightly before getting worse again, moderate (28.65%) and mild symptoms (49.54%) that increase over time, and patients who remain free of depression symptoms (15.51%) [19]. This study aims to provide further evidence on the outcomes of stroke survivors who have just returned to the community in an effort to better inform our understanding of development patterns of outcomes over time and identify those who are most in need of support.

Exploring sociodemographic and disease characteristics that contribute to patient-reported HRQoL, depression and anxiety symptoms can inform targeting of strategies that support recovery and adaptation to stroke [20]. Severity, type and location of stroke, access to acute medical and nursing care and stroke units, cognitive impairment, lifestyle factors [21] and presence of comorbidities [22] have all been related to long-term outcomes after a stroke [23]. At five years, depression, anxiety and disability were associated with lower HRQoL [12]. It has also been found that past or family history of stroke, aphasia and left hemisphere lesions can place survivors at-risk greater for mood disorders [15]. Investigations of predictors of both HRQoL and mood disorders among stroke survivors to guide service delivery in the early post-discharge phase are still lacking. Therefore, the aims of this study were to examine among a sample of stroke survivors: (1) self-reported physical and mental quality of life scores and level of depressive and anxiety symptoms 3 months post-hospital discharge; and (2) explore sociodemographic and clinical predictors of these early post-discharge outcomes.

## Methods

### Design

A cross-sectional survey was undertaken with stroke survivors recruited from eight hospitals located in New South Wales.

### Sample

Eligible stroke survivors were: admitted for stroke who were conscious and not requiring a high level of medical care; aged ≥ 18 years; ≤ 3 months post first ischemic or haemorrhagic stroke, or transient ischemic attack; being discharged home, or to private rehabilitation; and able to provide informed consent, as indicated by return of baseline survey. Survivors were ineligible if they (based on assessment by the hospital stroke care coordinator/ward staff) had: severe neurological impairment not associated with stroke; severe language or cognitive impairment; and/or insufficient English to complete patient-reported measures.

### Recruitment and data collection

Eligible stroke survivors discharged from participating hospitals were posted a study information and recruitment pack by clinic staff on behalf of the research team. Consenting survivors were asked to complete and return the survey to the researchers via a provided reply-paid envelope. A reminder pack was sent to all eligible survivors approximately two weeks after the initial information pack was mailed. Recruitment and data collection took place from April 2018 to December 2019.

### Measures

*Quality of life* was assessed using the Medical Outcomes Study Short Form version 2 (SF-12v2) [24, 25]. It measures 8 health concepts: physical functioning, role limitations due to physical health problems, bodily pain, general health, vitality, social functioning, role limitations, and mental health. Respondents indicate how often they have experienced problems in the last 4 weeks, with raw scores transformed to a 0 to 100 scale. Higher scores represent better health. It has adequate validity for measuring HRQoL among stroke survivors [26]. Scoring of the SF-12 items was performed using the QualityMetric PROCoRE Smart Measurement® System software.

*Depression* was assessed using the Patient Health Questionnaire 9 (PHQ-9; Cronbach’s α = 0.88) (9-items) [27]. This is a brief depression screening tool which has been used widely across a range of care settings and diagnostic groups. Higher scores indicate more severe depression. For the purposes of this study, a score of 10 or above was used to classify participants as depressed. A meta-analysis has shown that the tool has high specificity (0.79) and sensitivity (0.86) when used to screen and diagnose major depression [28].

*Anxiety* was assessed using The Generalized Anxiety Disorder Scale-7 (GAD-7; Cronbach’s α = 0.92) is a 7-item, self-rated scale [29]. Participants were asked to consider the preceding two weeks and to rate symptom frequency as not at all (0), several days (1), more than half of all days (2) or nearly all days (3). The GAD-7 has good reliability, and good criterion, factorial, and procedural validity [30]. Based on a recent meta-analysis, experts have recommended considering using a cut-off of 8 to optimize sensitivity without compromising specificity [30].

*Sociodemographic and clinical characteristics:* age, level of education attained, employment status, and type of stroke were obtained via standard self-report items. Treatment/s received was obtained (“What medical treatments did you receive at the time of your stroke?” with response options: “Thrombolysis (clot busting drug)”; “Carotid endarterectomy (surgery to remove plaque from the main artery in your neck)”; “Carotid angioplasty (surgery to place a stent in the main artery in your neck)”; “Endovascular clot retrieval (surgery to remove a clot from your brain)”; “Unsure”; and “Other”. Comorbid medical conditions were obtained by the item “Which of these health conditions apply to you?”, with response options: “History of Transient Ischaemic Attack (TIA)”; “Irregular Pulse (Atrial Fibrillation)”; “Diabetes”; “Fibromuscular Dysplasia (FMD)”; “High blood pressure”; “High cholesterol”; “Smoker”; “Obese (Body Mass Index above 30)”; “Overweight (Body Mass Index over 25)”; “Drink six or more standard alcoholic drinks per day”.

### Statistical analyses

Statistical analyses were programmed using SAS v9.4 (SAS Institute, Cary, North Carolina, USA). Descriptive statistics for categorical data are presented as count (%), or mean (SD) and median (min, max) if continuous. For participants with no more than 2 missing PHQ-9 items, missing values were imputed by mean imputation of non-missing responses. Mean imputation was performed on the GAD-7 where there were no more than 1 missing GAD-7 item per participant. Participants with 3 or more missing PHQ-9 item responses did not receive a total score for PHQ-9. Similarly, participants with 2 or more missing GAD-7 item responses did not receive a total score for GAD-7. Mean imputation is consistently used in previous works to address missing responses for PHQ-9 and GAD-7 [31–33]. Analyses of associations of sociodemographic factors, stroke type and treatment to the PCS and MCS scores of the SF-12 were performed with multiple linear mixed regression models; and to the PHQ-9 and GAD-7 severity categories (i.e. Minimal (0–4), Mild (5–9), Moderate (10–14), Moderately Severe (15–19) and Severe (20–27) Depression; and Minimal (0–4), Mild (5–9), Moderate (10–14), Severe (15–21) Anxiety) using ordinal logistic regression models. For these models, variables for sex, age, education, employment, BMI, type of stroke, indigenous status, health insurance and concession, smoking status, alcohol intake, and comorbidities (TIA, atrial fibrillation, diabetes, hypertension, and high cholesterol) were included as fixed effects. A random intercept for hospital site was included to account for correlated errors within clusters. Residual diagnostics on the linear mixed models did not suggest violations of linearity assumptions and model fit was deemed appropriate. The proportional odds assumption for ordinal logistic regression was verified by plotting the empirical cumulative logits of the outcomes for all explanatory variables and assessing each slope. Multiple imputation (MI) by fully conditional specification (FCS) was performed on missing sociodemographic and outcome observations, assuming missing observations were at random (MAR); due to 22% excluded observations during complete-case analysis, as analysis from FCS-MI has been shown to be produce estimates that are less biased than complete-case analysis [34–37]. The MI model was conditioned on predictor variables: BMI, employment status, health insurance, type of stroke, and co-morbidities; outcome variables: PCS, MCS, PHQ-9 score, and GAD-7 score; and auxiliary variables: stroke treatments, language other than English spoken at home, and change of employment due to stroke, to create m = 25 imputed data sets. Regressions for each outcome was performed on the m = 25 imputed data sets and estimates were combined using Rubin’s rules [38]. The pooled regression estimates are presented as mean differences with 95% confidence intervals (CI) for multiple linear regression models and odds ratios (OR) with 95% CI for ordinal regression models. A priori, *p* < 0.05 (two-tailed) was used to indicate statistical significance.

## Results

### Sample

A total of 1328 patients were assessed for study eligibility of which 1161 were identified as being eligible: adults with no or mild to moderate cognitive impairment, able to communicate in English, and discharged home or to rehabilitation from hospital. Reasons for ineligibility included: > 3 months post stroke (n = 3) and not discharged home (n = 165). Of the 1161 eligible patients who were posted a recruitment pack, 401 (35%) consented to participate and returned a completed survey. Table 1 presents the sociodemographic and clinical characteristics of participants.

**Table 1:** Sociodemographic and clinical characteristics of participants (N = 401)

Variable	Response	Total (N = 401)
Sex	Female	159 (40%)
	Male	235 (60%)
	Missing	7
Age	mean (SD)	71 (12)
	median (min, max)	73 (25, 100)
	18–64	99 (26%)
	≥ 65	279 (74%)
	Missing	23
Education	Secondary school or below	200 (52%)
	Trade or vocational training (e.g. TAFE or college)	131 (34%)
	Tertiary	57 (15%)
	Missing	13
Employment	Not working (home duties, unemployed, retired, disability pension)	312 (78%)
	Working (full, part time or casual)	89 (22%)
	Missing	0
Employment Status change because of stroke	No	332 (88%)
	Yes (please specify how below)	45 (12%)
	Missing	24
BMI	< 25	298 (78%)
	25–29	66 (17%)
	≥ 30	19 (5.0%)
	Missing	18
Treatment	None specified	42 (11%)
	Thrombolysis	110 (28%)
	Carotid endarterectomy/angioplasty or endovascular clot retrieval	38 (9.8%)
	Other	57 (15%)
	Unsure	142 (37%)
	Missing	12
Type of Stroke	Ischaemic stroke	157 (42%)
	Haemorrhagic stroke	20 (5.3%)
	Transient Ischaemic Attack	108 (29%)
	Unsure	93 (25%)
	Missing	23
Language other than English at home	No	344 (88%)
	Yes	46 (12%)
	Missing	11
Indigenous status	No	386 (98%)
	Yes, Aboriginal and/or Torres Strait Islander	6 (1.5%)
	Missing	9
Concession card	No	113 (29%)
	Yes	271 (71%)
	Missing	17
Health insurance	No	197 (51%)
	Yes	188 (49%)
	Missing	16
History of Transient Ischaemic Attack (TIA)	No	326 (85%)
	Yes	57 (15%)
	Missing	18
Irregular Pulse (Atrial Fibrillation)	No	294 (77%)
	Yes	89 (23%)
	Missing	18
Diabetes	No	279 (73%)
	Yes	104 (27%)
	Missing	18
Hypertension	No	176 (46%)
	Yes	207 (54%)
	Missing	18
High cholesterol	No	260 (68%)
	Yes	123 (32%)
	Missing	18
Smoker	No	333 (87%)
	Yes	50 (13%)
	Missing	18
Drink six or more standard alcoholic drinks per day	No	361 (94%)
	Yes	22 (5.7%)
	Missing	18

### SF-12v2 quality of life physical component score

Participants reported a mean SF-12v2 Physical Composite Score (PCS) quality of life score of 44.09 (SD = 9.57). Table 2 presents the pooled multiple linear regression estimates from MI (m = 25) data sets examining the relationship between sociodemographic and clinical factors and SF-12v2 PCS scores. Males scored on average 3.02 points higher (*p* = 0.001) on the PCS scale than females. Those who responded ‘Working’ scored on average 3.35 points higher (*p* = 0.030) on the PCS compared to those that responded’Not working’. Those that responded ‘Yes’ to having diabetes scored on average 4.48 points (*p* < 0.001) lower on the PCS scale than those that responded’No’ to having diabetes. Those that responded ‘Yes’ to having atrial fibrillation (AF) scored on average 2.71 points (*p* = 0.014) lower on the PCS scale than those that responded’No’.

**Table 2:** Factors associated with Quality of Life: Physical Component Score

Variable	Response	Mean difference (95% CI)	Contrast effect *p* value	Overall effect * p* value
Sex	Female	Ref	–	**0.001**
	Male	3.02 (1.17, 4.86)	**0.001**	
Age	18–64	Ref	–	0.645
	≥ 65	0.65 (− 2.10, 3.40)	0.645	
Education	Secondary school or below	Ref	–	0.589
	Trade or vocational training (e.g. TAFE or college	0.97 (− 1.03, 2.96)	0.342	
	Tertiary	1.05 (− 1.92, 4.02)	0.488	
Employment	Not working (home duties, unemployed, retired, disability pension)	Ref	–	**0.030**
	Working (full, part time or casual)	3.35 (0.33, 6.38)	**0.030**	
BMI	< 25	Ref	–	0.133
	25–29	− 0.78 (− 3.21, 1.66)	0.532	
	≥ 30	− 4.13 (− 8.24, − 0.01)	0.049	
Type of stroke	Ischaemic stroke	Ref	–	0.686
	Haemorrhagic stroke	2.14 (− 2.05, 6.33)	0.317	
	Transient Ischaemic Attack	− 0.29 (− 2.47, 1.89)	0.794	
	Unsure	− 0.47 (− 2.86, 1.91)	0.697	
Indigenous status	No	Ref	–	0.489
	Yes, Aboriginal and/or Torres Strait Islander	2.57 (− 4.70, 9.83)	0.489	
Concession card	No	Ref	–	0.112
	Yes	− 2.37 (− 5.30, 0.55)	0.112	
Health insurance	No	Ref	–	0.072
	Yes	1.78 (− 0.16, 3.73)	0.072	
History of Transient Ischaemic Attack (TIA)	No	Ref	–	0.275
	Yes	− 1.42 (− 3.96, 1.13)	0.275	
Irregular Pulse (Atrial Fibrillation)	No	Ref	**–**	**0.014**
	Yes	− 2.71 (− 4.87, − 0.54)	**0.014**	
Diabetes	No	Ref	–	**< 0.001**
	Yes	− 4.48 (− 6.57, − 2.38)	**< 0.001**	
Hypertension	No	Ref	–	0.173
	Yes	− 1.35 (− 3.30, 0.59)	0.173	
High cholesterol	No	Ref	–	0.518
	Yes	− 0.66 (− 2.67, 1.34)	0.518	
Smoker	No	Ref	–	0.428
	Yes	− 1.20 (− 4.17, 1.77)	0.428	
Drink six or more standard alcoholic drinks per day	No	Ref	–	0.863
	Yes	− 0.35 (− 4.36, 3.66)	0.863	

Values in bold indicate statistically significant associations

### SF-12 v2: mental component score

Participants reported a mean SF-12v2 Mental Composite Score (MCS) quality of life score of 46.84 (SD = 10.06). Table 3 presents the pooled multiple linear regression estimates from MI (m = 25) data sets exploring associations between sociodemographic and clinical factors and MCS scores. Those who reported a ‘history of TIA’ scored on average 3.22 (*p* = 0.030) points lower than those who did not have a history of TIA.

**Table 3:** Factors associated with baseline Quality of Life: Mental Component Score

Variable	Response	Mean difference (95% CI)	Contrast effect *p* value	Overall effect * p* value
Sex	Female	Ref	–	0.132
	Male	1.61 (− 0.48, 3.70)	0.132	
Age	18–64	Ref	–	0.299
	≥ 65	1.70 (− 1.51, 4.90)	0.299	
Education	Secondary school or below	Ref	–	0.222
	Trade or vocational training (e.g. TAFE or college	0.13 (− 2.14, 2.40)	0.911	
	Tertiary	2.85 (− 0.50, 6.20)	0.095	
Employment	Not working (home duties, unemployed, retired, disability pension)	Ref	–	0.224
	Working (full, part time or casual)	2.13 (− 1.31, 5.58)	0.224	
BMI	< 25	Ref	–	0.192
	25–29	− 2.46 (− 5.24, 0.32)	0.083	
	≥ 30	− 1.88 (− 6.64, 2.88)	0.439	
Type of stroke	Ischaemic stroke	Ref	–	0.661
	Haemorrhagic stroke	− 0.85 (− 5.55, 3.85)	0.724	
	Transient Ischaemic Attack	1.25 (− 1.32, 3.82)	0.341	
	Unsure	1.14 (− 1.60, 3.89)	0.413	
Indigenous status	No	Ref	–	0.283
	Yes, Aboriginal and/or Torres Strait Islander	− 4.54 (− 12.83, 3.75)	0.283	
Concession card	No	Ref	–	0.587
	Yes	0.93 (− 2.42, 4.27)	0.587	
Health insurance	No	Ref	–	0.219
	Yes	1.38 (− 0.82, 3.59)	0.219	
History of Transient Ischaemic Attack (TIA)	No	Ref	–	**0.030**
	Yes	− 3.22 (− 6.14, − 0.31)	**0.030**	
Irregular Pulse (Atrial Fibrillation)	No	Ref	–	0.560
	Yes	− 0.74 (− 3.23, 1.75)	0.560	
Diabetes	No	Ref	–	0.725
	Yes	− 0.43 (− 2.82, 1.97)	0.725	
Hypertension	No	Ref	–	0.086
	Yes	1.95 (− 0.28, 4.18)	0.086	
High cholesterol	No	Ref	–	0.674
	Yes	− 0.49 (− 2.78, 1.80)	0.674	
Smoker	No	Ref	–	0.120
	Yes	− 2.68 (− 6.07, 0.70)	0.120	
Drink six or more standard alcoholic drinks per day	No	Ref	–	0.363
	Yes	− 2.15 (− 6.80, 2.49)	0.363	

Values in bold indicate statistically significant associations

### Depression (PHQ-9)

The mean depression score on the PHQ-9 was 7.64 (SD = 6.48; range 0–27). Approximately one third of participants (34%, n = 132) were classified as depressed using a cut-off score of ≥ 10. Overall, 41% of survivors were classified as having minimal depression (PHQ-9 score ≤ 4; n = 160); 26% were classified as having mild depression (PHQ-9 score 5–9; n = 101); 18% were classified as having moderate depression (PHQ-9 score 10–14; n = 71); 9% were classified as having moderately severe depression (PHQ-9 score 15–19; n = 36) and 6% were classified as having severe depression (PHQ-9 score ≥ 20; n = 25). Table 4 presents the pooled ordinal logistic regression estimates from MI (m = 25) data sets showing characteristics associated with being in a lower PHQ-9 severity category. Males had approximately 2.11-fold higher odds (OR 2.11, *p* < 0.001) of being in a lower depression severity category compared to females. Participants with tertiary level education had approximately 2.52-fold higher odds (OR 2.52, *p* <  = 0.007) of having a lower depression severity compared to those with secondary school or below level of education. Participants with a history of TIA had approximately 54% lower odds (OR 0.46, *p* = 0.005) of being in a lower depression severity category compared to those with no history of TIA. Those with diabetes had approximately 45% lower odds (OR 0.55, *p* = 0.011) of being in a lower depression severity category than those without diabetes. Those who have 6 or more standard drinks of alcohol a day had approximately 62% lower odds (OR 0.38, *p* = 0.026) of being in a lower depression severity category than those who drink less than 6 standard drinks of alcohol a day.

**Table 4:** Factors associated with lower PHQ-9 depression severity

Variable	Response	Odds Ratio (95% CI)	Contrast effect * p* value	Overall effect * p* value
Sex	Female	Ref	–	**< 0.001**
	Male	2.11 (1.42, 3.13)	**< 0.001**	
Age	18–64	Ref	–	0.059
	≥ 65	1.80 (0.98, 3.33)	0.059	
Education	Secondary school or below	Ref	–	**0.011**
	Trade or vocational training (e.g. TAFE or college)	0.92 (0.60, 1.40)	0.701	
	Tertiary	2.52 (1.29, 4.94)	**0.007**	
Employment	Not working (home duties, unemployed, retired, disability pension)	Ref	–	0.216
	Working (full, part time or casual)	1.50 (0.79, 2.83)	0.216	
BMI	< 25	Ref	–	0.569
	25–29	0.80 (0.48, 1.34)	0.400	
	≥ 30	0.70 (0.28, 1.77)	0.456	
Type of stroke	Ischaemic stroke	Ref	–	0.633
	Haemorrhagic stroke	0.90 (0.37, 2.20)	0.815	
	Transient Ischaemic Attack	1.24 (0.76, 2.01)	0.393	
	Unsure	0.88 (0.52, 1.46)	0.612	
Indigenous status	No	Ref	–	0.286
	Yes, Aboriginal and/or Torres Strait Islander	0.37 (0.06, 2.28)	0.286	
Concession card	No	Ref	–	0.385
	Yes	1.33 (0.70, 2.53)	0.385	
Health insurance	No	Ref	–	0.128
	Yes	1.38 (0.91, 2.07)	0.128	
History of Transient Ischaemic Attack (TIA)	No	Ref	–	**0.005**
	Yes	0.46 (0.27, 0.80)	**0.005**	
Irregular Pulse (Atrial Fibrillation)	No	Ref	–	0.825
	Yes	0.95 (0.59, 1.52)	0.825	
Diabetes	No	Ref	–	**0.011**
	Yes	0.55 (0.35, 0.87)	**0.011**	
Hypertension	No	Ref	–	0.135
	Yes	1.38 (0.90, 2.10)	0.135	
High cholesterol	No	Ref	–	0.644
	Yes	1.11 (0.72, 1.71)	0.644	
Smoker	No	Ref	–	0.575
	Yes	0.84 (0.45, 1.56)	0.575	
Drink six or more standard alcoholic drinks per day	No	Ref	–	**0.026**
	Yes	0.38 (0.16, 0.89)	**0.026**	

Values in bold indicate statistically significant associations

### Anxiety (GAD-7)

The mean anxiety score was 4.82 (SD = 5.39; range 0–21), 27% of participants (n = 104) were classified as anxious using a cut-off score of ≥ 8; and 22% of participants (n = 80) were classified as “anxious” using a cut-off score of ≥ 10. Overall, sixty percent of survivors were classified as having minimal anxiety (GAD-7 score ≤ 4; n = 235); 19% were classified as having mild anxiety (GAD-7 score 5–9; n = 74); 13% were classified as having moderate anxiety (GAD-7 score 10–14; n = 51); and 7% were classified as having severe anxiety (GAD-7 score ≥ 15; n = 29). Table 5 presents the pooled ordinal logistic regression estimates from MI (m = 25) data sets showing characteristics associated with being in a lower anxiety severity category. Males had approximately 1.66-fold higher odds (OR 1.66, *p* = 0.022) of being in a lower anxiety severity category compared to females. Participants with a history of TIA had approximately 58% lower odds (OR 0.42, *p* = 0.003) of having a lower anxiety severity compared to those with no history of TIA.

**Table 5:** Factors associated with lower GAD-7 anxiety severity

Variable	Response	Odds ratio (95% CI)	Contrast effect * p* value	Overall effect * p* value
Sex	Female	Ref	–	**0.022**
	Male	1.66 (1.07, 2.56)	**0.022**	
Age	18–64	Ref	–	0.092
	≥ 65	1.75 (0.91, 3.37)	0.092	
Education	Secondary school or below	Ref	–	0.092
	Trade or vocational training (e.g. TAFE or college)	1.04 (0.65, 1.65)	0.872	
	Tertiary	2.32 (1.08, 4.98)	0.031	
Employment	Not working (home duties, unemployed, retired, disability pension)	Ref	–	0.259
	Working (full, part time or casual)	1.50 (0.74, 3.06)	0.259	
BMI	< 25	Ref	–	0.083
	25–29	0.56 (0.32, 0.96)	0.034	
	≥ 30	0.57 (0.20, 1.63)	0.292	
Type of stroke	Ischaemic stroke	Ref	–	0.569
	Haemorrhagic stroke	1.09 (0.39, 2.99)	0.873	
	Transient Ischaemic Attack	1.46 (0.85, 2.49)	0.171	
	Unsure	1.27 (0.72, 2.24)	0.402	
Indigenous status	No	Ref	–	0.098
	Yes, Aboriginal and/or Torres Strait Islander	0.24 (0.05, 1.30)	0.098	
Concession card	No	Ref	–	0.630
	Yes	1.19 (0.59, 2.38)	0.630	
Health insurance	No	Ref	–	0.108
	Yes	1.45 (0.92, 2.30)	0.108	
History of Transient Ischaemic Attack (TIA)	No	Ref	–	**0.003**
	Yes	0.42 (0.24, 0.75)	**0.003**	
Irregular Pulse (Atrial Fibrillation)	No	Ref	–	0.547
	Yes	1.18 (0.69, 1.99)	0.547	
Diabetes	No	Ref	–	0.430
	Yes	0.82 (0.51, 1.34)	0.430	
Hypertension	No	Ref	–	0.878
	Yes	1.04 (0.65, 1.65)	0.878	
High cholesterol	No	Ref	–	0.395
	Yes	1.23 (0.77, 1.97)	0.395	
Smoker	No	Ref	–	0.116
	Yes	0.59 (0.31, 1.14)	0.116	
Drink six or more standard alcoholic drinks per day	No	Ref	–	0.475
	Yes	0.72 (0.29, 1.78)	0.475	

Values in bold indicate statistically significant associations

## Discussion

This study examined the self-reported quality of life, depressive and anxiety symptoms among a sample of stroke survivors three months post-hospital discharge; and the sociodemographic and clinical predictors of these outcomes.

Our findings suggest that physical and mental quality of life scores of stroke survivors in our sample were lower than those reported in studies of the Australian general population [39]. Survivors in our sample who were female, not employed and had a comorbid diagnosis of diabetes or AF reported lower quality of life. Previous studies have found women have worse quality of life than men up to 12 months after stroke, even after adjusting for important sociodemographic variables and stroke severity [40]. Poorer quality of life of female stroke survivors has been attributed to older age, more severe strokes, greater pre-stroke dependency and post-stroke depression [41]. Despite this, interventions that address the specific disability and quality of life challenges for women following stroke are currently lacking [42].

Diabetes mellitus is an established risk factor for stroke and has been associated with poorer outcomes after stroke [43], including increased mortality, length of hospital stay, readmission rates, and poorer functional and rehabilitation outcomes after stroke [44]. Difficulties in self-care with diabetes may arise in the context of physical and cognitive stroke-related impairments [44]. For example, stroke survivors with hemiplegia may have reduced ability to self-administer insulin, cognitive demands of multiple medications may be high, and ability to participate in physical activity may be reduced [44]. Survivors in this study who were ‘not employed’ (including 86% of whom were retired and 12% whose employment had changed following stroke) reported lower physical component quality of life scores than those who were ‘employed’. A reduction in work activities [11] and reduced income [45] following stroke has been linked to poorer quality of life. As employment contributes to quality of life and satisfaction, these data reinforce the importance of identifying stroke patients of working age who may benefit from additional support to return to work [46]. A history of AF was also associated with lower physical component quality of life scores. AF is the most common heart rhythm disturbance globally and has previously been associated with increased risk of stroke and impaired quality of life [47].

Lower MCS was associated with a history of TIA. Recurrence of stroke after TIA or minor stroke has previously been associated with poor health related quality of life (HRQoL) and depression [48]. National stroke guidelines identify clinical conditions (hypertension, hyperlipidaemia, atrial fibrillation, diabetes, and obesity) and lifestyle factors (smoking, physical inactivity, unhealthy diet, and excess alcohol consumption) as significant modifiable risk factors that should be targeted for secondary prevention after an initial stroke or TIA [49]. More effective interventions to address unmet needs related to secondary prevention is a priority for stroke care [50]; as educational and behavioural interventions delivered in the absence of organisational change are not as effective in managing risks [51]. Where secondary prevention has not been effective and stroke has recurred, clinicians should be aware that people with recurrent stroke may be more at risk of poor mental health outcomes.

Our findings are consistent with previous literature which demonstrates that one third of stroke survivors are likely to experience post-stroke depression [14], with other mood disorders such as anxiety also prevalent (10% [15] to 27% [16]). Depression is associated with increased risk of mortality [14, 52], reduced social interaction [53, 54], and greater healthcare use [14, 55]. In spite of this, approximately two thirds of stroke patients are under-treated for depression [56].

Being male was associated with higher odds of having lower severity depression and anxiety, and having higher education was associated with higher odds of having lower severity depression, among our sample. This finding is consistent with a meta-analysis of longitudinal studies which showed that female gender is predictive of depression [57]. Having more than 8 years of education was protective factor, but only in the first three months’ post stroke. Similarly, female gender and being younger than 65 increased the risk of post stroke anxiety in a longitudinal study of over 2000 patients from a British stroke registry [58]. Depression has been reported to affect one in three survivors, and can interfere with a person’s ability to engage in post-stroke rehabilitation, resulting in in poorer recovery and quality of life [59]. Anxiety has been reported to affect between 32 and 38% of survivors [58]. Given the high rates of these outcomes, it is important for clinicians in the acute and rehabilitation settings to monitor for anxiety and depression. Whilst international guidelines differ on for whom, when and how screening for post-stroke depression and anxiety should occur [49, 60], our findings suggest that patients may benefit from increased psychological screening and support from clinicians and service providers after stroke, especially among women and those with a history of TIA.

### Implications for research, policy and practice

There may be benefits in offering additional support to those stroke survivors with clinical and/ or sociodemographic risk factors for poor psychosocial outcomes following stroke. For instance, our findings suggest that female stroke survivors may represent an important sub-group who may be in greater need of support in the early discharge phase, given their poorer quality of life and greater severity depression and anxiety. This is consistent with previous studies which have found that female survivors have poorer outcomes than males even up to 5 years post-stroke [61]. Further research examining the effectiveness of strategies to improve psychosocial outcomes of stroke survivors is urgently needed, given few studies have demonstrated efficacy of such interventions. Systematic reviews of information and communication technology interventions and psychosocial interventions for stroke populations have highlighted the limited benefits to date in achieving improvements in anxiety symptoms and quality of life of stroke survivors [62, 63]. Self-management programs, which attempt to actively involve survivors in managing their health, have shown promise in improving survivor health related quality of life [64, 65].

### Limitations

The recruitment of participants from multiple centres is a strength of this study. However, there are a number of limitations to the study. Only 35% of invited survivors completed a survey. As we did not have clinical and sociodemographic information about those who did not participate, it was not possible to assess responder bias. Further, we excluded those with severe cognitive impairment or aphasia limiting their ability to provide informed consent, or those survivors discharged to residential care. Therefore, the results of the study may not be generalizable to all stroke survivors.

## Conclusion

There are sub-groups of survivors who may be at-risk of poorer quality of life, psychological morbidity and a greater number of unmet needs in the early post-discharge phase. These findings represent an opportunity to improve the delivery of patient-centred care for stroke survivors, by supporting the identification and prioritisation of survivors who are most at-risk of poor outcomes in the early post-discharge phase and may therefore require modifications to standard hospital discharge processes, tailored to their level of risk.

## Data Availability

The datasets used and/or analysed during the current study are available from the corresponding author on reasonable request.
